# Intakes of Specific Categories of Vegetables and Fruits Are Inversely Associated With Depressive Symptoms Among Adults

**DOI:** 10.2188/jea.JE20200003

**Published:** 2021-03-05

**Authors:** Jing Sun, Zhaoying Li, Yan Li, Dongfeng Zhang

**Affiliations:** Department of Epidemiology and Health Statistics, the School of Public Health of Qingdao University, Shandong, China

**Keywords:** adult, cross-sectional studies, depression, fruit, vegetables

## Abstract

**Background:**

Epidemiological evidence on the relationships between intakes of different categories of vegetables and fruits and depressive symptoms is very limited and inconsistent, especially with no evidence from the general population. This study aimed to estimate their relationships among a large general population.

**Methods:**

The cross-sectional design was based on the National Health and Nutrition Examination Survey (2007–2014) and included 16,925 adults. Dietary information was attained from two nonconsecutive 24-hr dietary recalls. Patient Health Questionnaire was applied for measuring depressive symptoms. The associations between vegetables and fruits intakes and depressive symptoms were appraised utilizing logistic regression and restricted cubic spline.

**Results:**

Compared with the lowest category of intake, the most-adjusted odds ratios of depressive symptoms for the highest intake category of tomatoes and tomato mixtures were 0.81 (95% confidence interval [CI], 0.66–0.99), and 0.64 (95% CI, 0.48–0.85) for dark-green vegetables, 0.67 (95% CI, 0.53–0.84) for other vegetables, 0.48 (95% CI, 0.29–0.79) for berries, 0.67 (95% CI, 0.55–0.82) for total vegetables, and 0.70 (95% CI, 0.57–0.86) for total fruits, and for the medium categories of bananas and dried fruits were 0.62 (95% CI, 0.41–0.95) and 0.39 (95% CI, 0.19–0.81), respectively. After sensitivity analysis further excluding subjects with co-morbid health conditions, these findings remained significant, except for bananas. An L-shaped relationship was observed between depressive symptoms and intake of total vegetables, while the association was linear with total fruits intake.

**Conclusions:**

Intakes of tomatoes and tomato mixtures, dark-green vegetables, other vegetables, berries, dried fruits, total vegetables, and total fruits were inversely related to depressive symptoms among adults.

## INTRODUCTION

As important constituents of diet, vegetable and fruit abound with dietary fiber, phytochemicals, magnesium, folate, vitamin C, vitamin B2, vitamin B6, and other nutrients.^1^ Therefore, they may have a large number of health benefits. Many epidemiologic studies have been carried out to explore the relationships of vegetable and fruit intakes with diseases, and results have indicated that intakes of vegetable and fruit were inversely associated with the risks of cardiovascular disease, total cancer,^2^ all-cause mortality,^2^^,^^3^ metabolic syndrome,^4^ cognitive disorders,^5^ inflammatory bowel disease,^6^ and hypertension.^7^ According to the Global Burden of Disease Study, low consumption of fruits was one of the primary dietary risk factors, resulting in 65 million disability-adjusted life years as well as two million deaths globally in 2017.^8^

Recently, there is a persistent interest in evaluating the associations between vegetable and fruit intakes and depression.^9^^,^^10^ Although the etiology of depression is not completely known, studies have shown that it is closely related to the activation of the inflammatory response system and oxidative stress.^11^^–^^13^ Owing to the anti-inflammatory and antioxidant characteristics of vegetables and fruits,^14^^,^^15^ they may play an important role in protecting against depression. A meta-analysis revealed that vegetables and fruits intakes were negatively associated with depression.^16^

Meanwhile, the main nutrient components of different vegetable and fruit categories are diverse.^1^^,^^17^ A prospective cohort study has found that the relationships with type 2 diabetes were heterogeneous among different types of fruits due to variable nutrient components of them.^18^ Similarly, the associations between different categories of vegetables (or fruits) and cancer were also discrepant.^19^ Furthermore, different categories of vegetables or fruits differed in component and content of nutrients causing highly varied antioxidant and anti-inflammatory capacities among them, for example, berries have the highest antioxidant activity in common fruits due to its high phenolic content.^15^^,^^20^^,^^21^ Thus, different types of vegetables and fruits may also have an unequal effect on depression, and it is necessary to explore the relationships with depression by fruit and vegetable types. Several studies have evaluated the associations of depression with different kinds of vegetables or fruits intakes; however, these were limited to particular group of people (young adults, older people, or females), and the results are inconsistent.^22^^–^^25^ Especially, their associations in the general population are still completely unknown. Besides, the dose-response relationship of total vegetables or fruits with depression risk was also unclear. Hence, the purpose of the current study utilizing data based on the National Health and Nutrition Examination Survey (NHANES) 2007–2014 was to estimate relationships of intakes of different categories of vegetables or fruits, as well as total vegetables or fruits, with depressive symptoms risk among the large general population. Additionally, the dose-response relationship between total vegetables or fruits consumption and depressive symptoms was also assessed in this study.

## MATERIAL AND METHODS

### Study population

NHANES is a complex, stratified, multi-stage, and cross-sectional probability sampling design, and the sample of NHANES represents nationally non-institutionalized American civilians.^26^ In order to measure the nutrition condition and health status of Americans, the data of NHANES have been collected via household interviews and examinations implemented by the mobile examination center (MEC) in 2-year cycles after 1999. Informed consent was provided by all subjects, and investigation protocol was authorized via the Research Ethics Review Board of the National Center for Health Statistics.^27^

**Figure 1.**
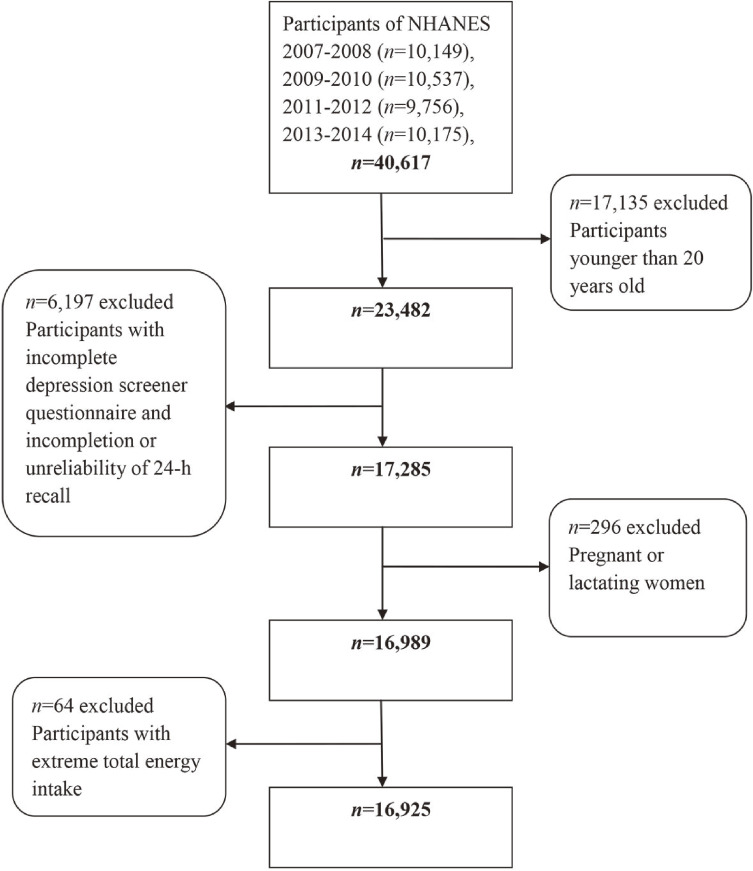
Flowchart of the screening process for selecting eligible individuals from NHANES 2007–2014. NHANES, National Health and Nutrition Examination Survey.

The present study combined four-cycle data from NHANES (2007–2008, 2009–2010, 2011–2012, and 2013–2014). A total of 40,617 participants were included in the 2007–2014 NHANES and this study chose 23,482 individuals (age ≥20 years). Of these, participants with incomplete depression screener questionnaire (*n* = 3,351), and incompletion or unreliability of 24-hr recall (*n* = 2,846) were omitted. Individuals who were pregnant (*n* = 188) and lactating women (*n* = 108) were left out. Furthermore, subjects with extreme total energy intake (males: less than 500 or more than 8,000 kcal/day, females: less than 500 or more than 5,000 kcal/day) were excluded (*n* = 64). Finally, the present study included 16,925 subjects aged 20 years and over (8,326 males and 9,418 females) (Figure 1).

### Depressive symptoms assessment

NHANES used the nine-item Patient Health Questionnaire (PHQ-9) to measure depressive symptoms. It is a valid instrument for detecting depressive symptoms.^28^ Each of the 9 items is scored from 0 to 3, thus the total score ranges from 0 to 27. Participants with the total score equal to 10 or higher were classified as depressive symptoms group.^29^

### Dietary vegetable and fruit assessment

Dietary information was attained from two nonconsecutive 24-hr dietary recall interviews. The recall data were obtained in person on the MEC for the first interview and via telephone after 3–10 days for the second, respectively. Validation studies have demonstrated the accuracy of the dietary information, and the correlation coefficient of total energy intake using 24-hr dietary recall with total energy expenditure measured by doubly labeled water is 0.53 (*P* = 0.02).^30^^,^^31^ Nutrient contents and food were counted based on this.^32^ Intakes of different types of vegetables or fruits and total vegetables or fruits were identified according to food codes. In this study, vegetable categories included white potatoes and Puerto Rican starchy, tomatoes and tomato mixtures, deep-yellow vegetables, dark-green vegetables, and other vegetables; and fruit categories included apples, bananas, berries, dried fruits, citrus, and melons in this study (eTable 1). More details of vegetable and fruit categories are available online.^32^ Dietary vegetables and fruits intakes were calculated using the two 24-hr dietary interviews to obtain the mean intakes.

### Covariates

According to prior literature,^33^^–^^36^ demography variables (age, gender, race, marital status, educational level, and annual family income), lifestyle factors (work physical activity, recreational physical activity, alcohol consumption, smoking status, and total daily energy intake), body mass index (BMI), and co-morbid health conditions (hypertension, diabetes, heart disease, and stroke) were chosen as covariates to control potential confounding effects. The classifications and criteria of covariates are presented in eTable 2.

### Statistical analysis

Statistical analyses were carried out utilizing Stata 15.0 (Stata Corp, College Station, TX, USA)^37^ to account for the complexity of sampling design. Strata information, primary sampling unit, and weights were used in current analyses for generating nationally representative estimation. The study combined four cycles of NHANES (from 2007 to 2014), so new 8-year dietary weights were calculated and utilized in the analyses.^38^

In the present study, quantitative data were described as medians and inter-quartile ranges due to their non-normal distributions based on the Kolmogorov-Smirnov normality test. Qualitative data were expressed as numbers and percentages. For non-normal continuous variables, Mann-Whitney *U* tests were utilized for comparing averages between depressive symptoms group and non-depressive symptoms group. For categorical variables, chi-square tests were utilized for comparing percentages in the two groups. Total vegetables and total fruits intakes were divided into tertiles according to their distributions in the current entire study population. Since a minority of subjects consuming the single specific category of vegetable or fruit, each type of vegetable or fruit intake was segmented into three categories. Participants with no consumption of the specific category of vegetable or fruit were classified into group 1 (intake = 0), and individuals with consumption were divided into group 2 (<median) and group 3 (≥median) based on its median intake (g/d) among the participants with consumption. Associations of intakes of different types of vegetables or fruits, as well as total vegetables or fruits, with depressive symptoms were appraised using logistic regression analyses, with the lowest intake category as the referent. In model 1, covariates included gender, age, educational level, race, marital status, family income, BMI, alcohol consumption, smoking status, recreational physical activity, work physical activity, total energy intake (continuous, kcal/d), diabetes, hypertension, heart disease, and stroke. Model 2 additionally adjusted for total vegetables intake (or total fruits intake) (g/d) to assess the independent associations between subgroups of vegetables (or fruits) and depressive symptoms. Moreover, stratified analyses were conducted by age and gender, respectively. Given that participants with a history of diabetes, heart disease, or stroke might be forced to improve lifestyle, we also performed a sensitivity analysis by further excluding subjects with these diseases of doctor diagnosis. In order to appraise the dose-response relationship of total vegetables or fruits intake with depressive symptoms, a restricted cubic spline with three knots (the 5th, 50th, and 95th percentiles of exposure distribution) was applied in the model 1. The values of *P* for nonlinearity were estimated via testing whether the coefficients of the second splines were equal to 0 or not. Linear trend tests were carried out via logistic regression, and using the median of every exposure intake category formed a new ordinal variable. A *P* value (two-sided) below 0.05 was defined as statistically significant level.

## RESULTS

Table 1 displays the characteristics of the study participants across depressive symptoms on the basis of the PHQ-9 score. A total of 16,925 eligible participants were included, and 49.2% of them were males. The prevalence of depressive symptoms among the current population was 9.4%. These characteristics between depressive symptoms and non-depressive symptoms group were significantly different except for alcohol consumption status. Depressive symptoms were inclined to the obese individuals, smokers, younger, females, individuals with lower family income, less education level, lower recreational physical activity and work physical activity levels, lower total daily dietary energy, diabetes, hypertension, heart disease and stroke in this sample. In correlation analyses, all the spearman’s correlation coefficients between the first and second 24-hr dietary recalls for total vegetables or fruits or different types of them intakes were significant (*P* < 0.001) (eTable 3).

**Table 1.** Characteristics of participants aged 20 or older by depressive symptoms, National Health and Nutrition Examination Survey 2007–2014

Characteristics	Without depressive symptoms (PHQ <10)	With depressive symptoms (PHQ ≥10)	*P* value^a^
**Number of subjects (%)**	15,332 (90.6)	1,593 (9.4)	
**Age, years (%)**			<0.001
20–39	4,825 (31.5)	476 (29.9)	
40–59	5,070 (33.1)	675 (42.4)	
≥60	5,437 (35.5)	442 (27.7)	
**Gender (%)**			<0.001
Male	7,766 (50.7)	560 (35.2)	
Female	7,566 (49.3)	1,033 (64.8)	
**Race (%)**			<0.001
Mexican American	2,159 (14.1)	231 (14.5)	
Other Hispanic	1,457 (9.5)	216 (13.6)	
Non-Hispanic White	7,211 (47.0)	707 (44.4)	
Non-Hispanic Black	3,180 (20.7)	341 (21.4)	
Other race	1,325 (8.6)	98 (6.2)	
**Marital status (%)**			<0.001
Married/living with partner	9,344 (61.0)	723 (45.4)	
Widowed/divorced/separated/never married	5,980 (39.0)	869 (54.6)	
**Educational level (%)**			<0.001
Below high school	3,451 (22.5)	587 (36.8)	
High school	3,521 (23.0)	377 (23.7)	
Above high school	8,347 (54.5)	629 (39.5)	
**Family income (%)**			<0.001
Under $20,000	3,317 (22.4)	676 (44.6)	
$20,000 and over	11,459 (77.6)	839 (55.4)	
**Body mass index (BMI) (%)**			<0.001
<25 kg/m^2^	4,369 (28.7)	365 (23.2)	
25 to <30 kg/m^2^	5,204 (34.2)	414 (26.3)	
≥30 kg/m^2^	5,629 (37.0)	794 (50.5)	
**Work physical activity (%)**			0.001
Vigorous	2,836 (18.5)	278 (17.5)	
Moderate	3,347 (21.8)	289 (18.2)	
Other	9,147 (59.7)	1,024 (64.4)	
**Recreational physical activity (%)**			<0.001
Vigorous	3,346 (21.8)	157 (9.9)	
Moderate	4,318 (28.2)	312 (19.6)	
Other	7,668 (50.0)	1,124 (70.6)	
**Smoked at least 100 cigarettes in a lifetime (%)**	6,750 (44.0)	937 (58.8)	<0.001
**Had at least 12 alcohol drinks one year (%)**	11,156 (72.8)	1,157 (72.8)	>0.99
**Hypertension (%)**	7,756 (51.1)	849 (54.1)	0.026
**Diabetes (%)**	2,730 (18.1)	419 (26.9)	<0.001
**Heart disease (%)**	1,208 (7.9)	247 (15.6)	<0.001
**Stroke (%)**	511 (3.3)	119 (7.5)	<0.001
**Total energy intake (kcal/d), median (interquartile range)**	1,912.3 (995.3)	1,801.0 (993.8)	<0.001

PHQ, Patient Health Questionnaire.

^a^*P* values derived from chi-square tests for categorical variables and Mann-Whitney *U* tests for non-normal continuous variables.

The results from logistic regression model of depressive symptoms across different categories of vegetables and total vegetables intakes are presented in Table 2. After multivariate adjustment (model 1), there were still significantly negative associations of tomatoes and tomato mixtures, deep-yellow vegetables, dark-green vegetables, other vegetables, and total vegetables intakes with depressive symptoms, and the odds ratio (OR) of depressive symptoms for the highest category compared with the lowest category of total vegetables was 0.67 (95% confidence interval [CI], 0.55–0.82). In model 2, which was additionally adjusted for total vegetables intake, the correlation of deep-yellow vegetables with depressive symptoms was no longer significant, while associations of tomatoes and tomato mixtures, dark-green vegetables, and other vegetables intakes with depressive symptoms remained significant, and the highest category ORs were 0.81 (95% CI, 0.66–0.99), 0.64 (95% CI, 0.48–0.85), and 0.67 (95% CI, 0.53–0.84), respectively.

**Table 2.** Weighted odds ratios (95% confidence intervals) for depressive symptoms across different categories of vegetables and total vegetables intakes, National Health and Nutrition Examination Survey 2007–2014

	Cases/participants	Crude	Model 1^a^	Model 2^b^
**White potatoes and Puerto Rican starchy vegetables intake (g/d)^c^**				
Group 1 (=0)	659/7,191	1.00 (reference)	1.00 (reference)	1.00 (reference)
Group 2 (<96.97)	478/4,867	1.01 (0.83–1.25)	1.02 (0.83–1.26)	1.02 (0.83–1.25)
Group 3 (≥96.97)	456/4,867	0.98 (0.80–1.20)	0.96 (0.76–1.21)	1.03 (0.81–1.32)
*P*-trend^d^		0.824	0.691	0.800
**Tomatoes and tomato mixtures intake (g/d)^c^**				
Group 1 (=0)	752/6,856	1.00 (reference)	1.00 (reference)	1.00 (reference)
Group 2 (<40.00)	459/4,801	0.74 (0.61–0.90)^**^	0.82 (0.67–1.00)	0.83 (0.68–1.01)
Group 3 (≥40.00)	382/5,268	0.59 (0.50–0.69)^***^	0.77 (0.64–0.92)^**^	0.81 (0.66–0.99)^*^
*P*-trend^d^		<0.001	0.013	0.067
**Deep-yellow vegetables intake (g/d)^c^**				
Group 1 (=0)	1,391/13,689	1.00 (reference)	1.00 (reference)	1.00 (reference)
Group 2 (<51.25)	106/1,615	0.72 (0.50–1.05)	0.89 (0.58–1.35)	0.91 (0.59–1.40)
Group 3 (≥51.25)	96/1,621	0.52 (0.38–0.71)^***^	0.69 (0.50–0.96)^*^	0.73 (0.53–1.02)
*P*-trend^d^		<0.001	0.029	0.069
**Dark-green vegetables intake (g/d)^c^**				
Group 1 (=0)	1,392/13,358	1.00 (reference)	1.00 (reference)	1.00 (reference)
Group 2 (<78.00)	103/1,776	0.56 (0.43–0.74)^***^	0.66 (0.47–0.93)^*^	0.67 (0.47–0.95)^*^
Group 3 (≥78.00)	98/1,791	0.51 (0.37–0.69)^***^	0.61 (0.46–0.81)^**^	0.64 (0.48–0.85)^**^
*P*-trend^d^		<0.001	<0.001	0.001
**Other vegetables intake (g/d)^c^**				
Group 1 (=0)	499/3,877	1.00 (reference)	1.00 (reference)	1.00 (reference)
Group 2 (<89.86)	608/6,524	0.66 (0.55–0.80)^***^	0.79 (0.63–0.99)^*^	0.80 (0.63–1.00)
Group 3 (≥89.86)	486/6,524	0.48 (0.40–0.57)^***^	0.65 (0.53–0.79)^***^	0.67 (0.53–0.84)^**^
*P*-trend^d^		<0.001	0.001	0.005
**Total vegetables intake (g/d)^e^**				
Tertile 1 (<106.47)	687/5,641	1.00 (reference)	1.00 (reference)	
Tertile 2 (106.47 to <226.56)	490/5,641	0.65 (0.53–0.79)^***^	0.74 (0.58–0.93)^*^	
Tertile 3 (≥226.56)	416/5,643	0.52 (0.44–0.62)^***^	0.67 (0.55–0.82)^***^	
*P*-trend^d^		<0.001	<0.001	

^a^Model 1 adjusted for gender, age, race, marital status, educational level, family income, body mass index, recreational physical activity, work physical activity, smoking status, alcohol consumption, hypertension, diabetes, heart disease, stroke, and total daily energy intake (continuous, kcal/d).

^b^Model 2 additionally adjusted for total vegetables intake (continuous, g/d) for subgroups of vegetables.

^c^Each type of vegetable intake was segmented into three categories. Participants with no consumption the specific category of vegetable were classified into group 1 (intake = 0), and individuals with consumption were divided into group 2 (<median) and group 3 (≥median) based on its median intake (g/d) among the participants with consumption.

^d^Tests for linear trend were carried out by logistic regression, using a median value of each exposure intake category as a single ordinal variable.

^e^Total vegetables intake was divided into tertiles according to its distribution in the current entire study population.

^*^P < 0.05; ^**^P < 0.01; ^***^P < 0.001.

Table 3 shows the associations of different types of fruits and total fruits intakes with depressive symptoms. In multivariate adjustment model 1, intakes of berries, bananas, dried fruits, and total fruits still significantly negatively related to depressive symptoms, and the OR of depressive symptoms for the highest category of total fruits was 0.70 (95% CI, 0.57–0.86). After additional adjustment total fruits intake, the inverse correlations between these subgroups of fruits and depressive symptoms did not change substantially, and the ORs were 0.48 (95% CI, 0.29–0.79) for the highest category of berries and 0.62 (95% CI, 0.41–0.95) and 0.39 (95% CI, 0.19–0.81) for the medium categories of bananas and dried fruits, respectively.

**Table 3.** Weighted odds ratios (95% confidence intervals) for depressive symptoms across different categories of fruits and total fruits intakes, National Health and Nutrition Examination Survey 2007–2014

	Cases/participants	Crude	Model 1^a^	Model 2^b^
**Apples intake (g/d)^c^**				
Group 1 (=0)	1,346/13,549	1.00 (reference)	1.00 (reference)	1.00 (reference)
Group 2 (<182.00)	121/1,487	0.82 (0.60–1.10)	0.81 (0.57–1.14)	0.84 (0.60–1.19)
Group 3 (≥182.00)	126/1,889	0.63 (0.46–0.86)^**^	0.75 (0.51–1.12)	0.81 (0.54–1.21)
*P*-trend^d^		0.003	0.080	0.183
**Bananas intake (g/d)^c^**				
Group 1 (=0)	1,274/12,394	1.00 (reference)	1.00 (reference)	1.00 (reference)
Group 2 (<118.00)	85/1,390	0.54 (0.38–0.78)^**^	0.60 (0.40–0.90)^*^	0.62 (0.41–0.95)^*^
Group 3 (≥118.00)	234/3,141	0.77 (0.60–0.97)^**^	1.01 (0.78–1.31)	1.08 (0.84–1.39)
*P*-trend^d^		0.005	0.529	0.902
**Berries intake (g/d)^c^**				
Group 1 (=0)	1,499/15,020	1.00 (reference)	1.00 (reference)	1.00 (reference)
Group 2 (<67.00)	44/951	0.41 (0.28–0.60)^***^	0.72 (0.48–1.07)	0.73 (0.49–1.09)
Group 3 (≥67.00)	50/954	0.33 (0.21–0.50)^***^	0.45 (0.27–0.75)^**^	0.48 (0.29–0.79)^**^
*P*-trend^d^		<0.001	0.001	0.003
**Dried fruits intake (g/d)^c^**				
Group 1 (=0)	1,529/15,913	1.00 (reference)	1.00 (reference)	1.00 (reference)
Group 2 (<25.50)	24/505	0.35 (0.19–0.64)^**^	0.38 (0.18–0.80)^*^	0.39 (0.19–0.81)^*^
Group 3 (≥25.50)	40/507	0.56 (0.34–0.92)^*^	0.89 (0.54–1.48)	0.92 (0.55–1.53)
*P*-trend^d^		0.010	0.348	0.414
**Citrus intake (g/d)^c^**				
Group 1 (=0)	1,441/14,790	1.00 (reference)	1.00 (reference)	1.00 (reference)
Group 2 (<131.00)	56/881	0.53 (0.35–0.78)^**^	0.65 (0.42–1.00)	0.67 (0.43–1.03)
Group 3 (≥131.00)	96/1,254	0.86 (0.63–1.18)	0.97 (0.66–1.44)	1.06 (0.71–1.58)
*P*-trend^d^		0.048	0.459	0.747
**Melons intake (g/d)^c^**				
Group 1 (=0)	1,491/15,464	1.00 (reference)	1.00 (reference)	1.00 (reference)
Group 2 (<152.00)	45/715	0.73 (0.42–1.27)	1.00 (0.53–1.90)	1.04 (0.55–1.99)
Group 3 (≥152.00)	57/746	0.66 (0.43–1.01)	0.76 (0.48–1.20)	0.87 (0.53–1.43)
*P*-trend^d^		0.031	0.250	0.620
**Total fruits intake (g/d)^e^**				
Tertile 1 (<78.08)	688/5,641	1.00 (reference)	1.00 (reference)	
Tertile 2 (78.08 to <249.00)	479/5,596	0.67 (0.53–0.85)^**^	0.79 (0.60–1.05)	
Tertile 3 (≥249.00)	426/5,688	0.53 (0.44–0.64)^***^	0.70 (0.57–0.86)^*^	
*P*-trend^d^		<0.001	0.001	

^a^Model 1 adjusted for gender, age, race, marital status, educational level, family income, body mass index, recreational physical activity, work physical activity, smoking status, alcohol consumption, hypertension, diabetes, heart disease, stroke, and total daily energy intake (continuous, kcal/d).

^b^Model 2 additionally adjusted for total fruits intake (continuous, g/d) for subgroups of fruits.

^c^Each type of fruit intake was segmented into three categories. Participants with no consumption the specific category of fruit were classified into group 1 (intake = 0), and individuals with consumption were divided into group 2 (<median) and group 3 (≥median) based on its median intake (g/d) among the participants with consumption.

^d^Tests for linear trend were carried out by logistic regression, using a median value of each exposure intake category as a single ordinal variable.

^e^Total fruits intake was divided into tertiles according to its distribution in the current entire study population.

^*^P < 0.05; ^**^P < 0.01; ^***^P < 0.001.

The relationships of different types of vegetables and fruits and total of them with depressive symptoms in stratified analyses across gender and age are presented in eTable 4 and eTable 5, respectively. For total vegetables and total fruits, both of them were inversely related to depressive symptoms in all gender and age groups, except for age group (20 to 39 years) which only total vegetables intake was significant. For different types of vegetables, in addition to those types showing significant associations in unstratified analyses, other types also showed significantly negative association with depressive symptoms, such as deep-yellow vegetables in the 20–39 years age group and the ≥60 years age group, bananas in females, citrus in the 40–59 years age group, and melons in the 20–39 years age group.

**Figure 2.**
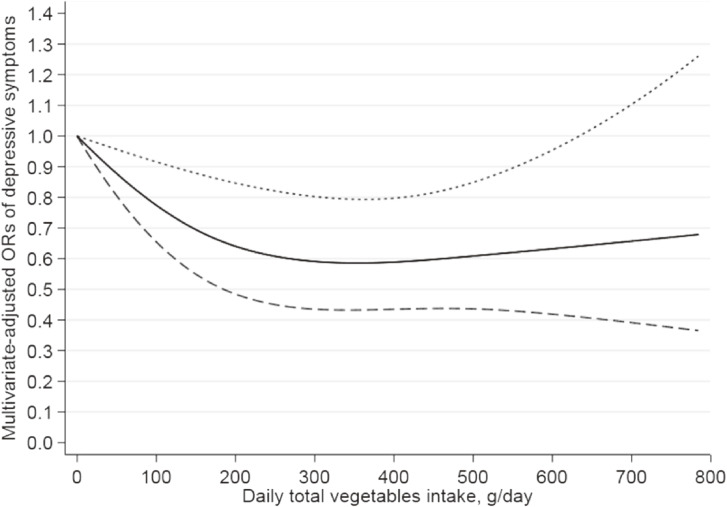
Restricted cubic spline model of the odds ratios (ORs) of depressive symptoms with total vegetables intake. The solid line and dashed lines represent the estimated ORs and the 95% confidence intervals. The lowest level of total vegetables intake (0 g/d) was as the referent. The relationship adjusted for gender, age, race, marital status, educational level, family income, BMI, recreational physical activity, work physical activity, smoking status, alcohol consumption, hypertension, diabetes, heart disease, stroke, and total daily energy intake (continuous, kcal/d). BMI, body mass index.

**Figure 3.**
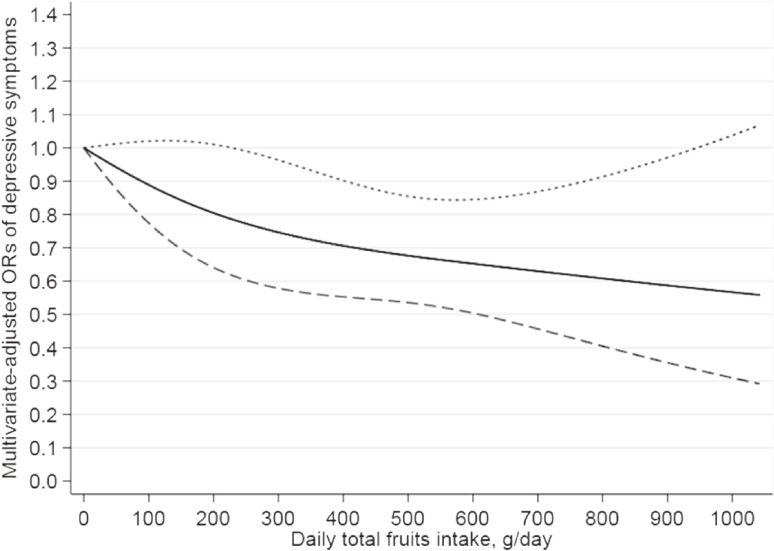
Restricted cubic spline model of the odds ratios (ORs) of depressive symptoms with total fruits intake. The solid line and dashed lines represent the estimated ORs and the 95% confidence intervals. The lowest level of total fruits intake (0 g/d) was as the referent. The relationship adjusted for gender, age, race, marital status, educational level, family income, BMI, recreational physical activity, work physical activity, smoking status, alcohol consumption, hypertension, diabetes, heart disease, stroke, and total daily energy intake (continuous, kcal/d). BMI, body mass index.

The dose-response relationships between total vegetables and total fruits intakes and the risk of depressive symptoms were estimated by restricted cubic spline analyses, and results are depicted in Figure 2 and Figure 3, respectively. The nonlinear inverse (L-shaped) relationship was observed between total vegetables intake and depressive symptoms (*P* for nonlinearity = 0.04). When intake arrived to 100, 200, 300, and 400 g/d, the ORs were 0.77 (95% CI, 0.65–0.92), 0.64 (95% CI, 0.48–0.85), 0.59 (95% CI, 0.44–0.80), and 0.59 (95% CI, 0.43–0.80), respectively (Figure 2). There was a linear inverse relationship between total fruits consumption and depressive symptoms (*P* for nonlinearity = 0.49). When total fruits consumption reached around 240 g/d (OR, 0.78; 95% CI, 0.61–0.99), it presented a significantly inverse association with depressive symptoms. When intake reached 300, 500, 700, and 900 g/d, the ORs were 0.75 (95% CI, 0.58–0.96), 0.68 (95% CI, 0.54–0.85), 0.63 (95% CI, 0.46–0.87), and 0.59 (95% CI, 0.36–0.97), respectively (Figure 3). The restricted cubic spline adjusted multiple confounders, which were consistent with logistic regression model 1.

The weighted ORs of depressive symptoms across different categories of vegetables and fruits and total of them after removing 3,410 individuals with co-morbid health conditions (diabetes/heart disease/stroke) are displayed in eTable 6. Except for bananas, other findings did not change substantially.

## DISCUSSION

The current study used data from NHANES (2007–2014) including 16,925 American participants aged 20 years or older to evaluate the associations of different categories of vegetables or fruits and total vegetables or fruits intakes, with the risk of depressive symptoms. The results indicated that intakes of tomatoes and tomato mixtures, dark-green vegetables, other vegetables, berries, dried fruits, total vegetables, and total fruits were negatively related to depressive symptoms, even after adjustment for multiple potential confounders and sensitivity analysis excluding subjects with co-morbid health conditions. In stratified analyses across gender and age, besides the above-mentioned categories, negative associations were also observed between other types (deep-yellow vegetables, bananas, citrus, and melons) and depressive symptoms. As far as we know, this study is the first assessment of the associations of different categories of vegetables and fruits, total vegetables, and total fruits intakes with depressive symptoms, with a dose-response relationship among the general population.

Thus far, some epidemiological studies have evaluated the associations of total vegetables and total fruits intakes with depressive symptoms. Among them, a meta-analysis including eight studies for vegetable intake and ten studies for fruit intake has demonstrated that both vegetables and fruits intakes were negatively related to depression,^16^ which was consistent with this current result.

In respect of different types of vegetables, a cross-sectional study among young adults aged 18–25 years^23^ and another among Iranian women aged 20–49 years^22^ revealed that dark leafy greens consumption was negatively associated with depressive symptoms or depression, which were consistent with this study. However, a cross-sectional study conducted in older Japanese indicated that green-leaf vegetables intake was not statistically related to depressive symptoms.^24^ The negative relationship of tomatoes/tomato products intake with depressive symptoms was presented in the study,^24^ whereas the association was not significant in another study.^23^ A prospective cohort among Italian participants revealed that no statistical association between potatoes consumption and depressive symptoms,^39^ which was in line with the present study finding. For deep-yellow vegetables, the study suggested that intake of carrots (one of the dark yellow vegetables) was inversely associated with depressive symptoms among young adults,^23^ and the inverse association was also observed among subjects aged 20–39 years in this study. By contrast, another study showed no significant association of dark yellow vegetables with depressive symptoms,^22^ and our result also showed no significance in the whole population. Further studies are needed to appraise their relationship in different age groups, and mechanism researches are required to explain the difference.

As for different categories of fruits, similarly to this study result, cross-sectional studies suggested that berries intake was negatively related to depression^22^ or depressive symptoms.^23^ However, a follow-up study among midlife and older women found no statistically significant association between intakes of strawberries and blueberries and depression.^25^ Studies reported that citrus consumption was negatively associated with depression,^22^^,^^25^^,^^40^ and the negative correlation was also found among individuals aged 40–59 years in our study. Different from this study finding, the study suggested that apple intake was inversely related to depression.^23^ For bananas, the study found a negative association between bananas intake and depressive symptoms,^23^ and we also observed the negative correlation, whereas it was no longer significant after removing individuals with co-morbid health conditions. A recent cross-sectional study in Chinese adults reported that moderate banana intake was inversely related to depressive symptoms among males, whereas high banana intake (≥4 times/week) was positively associated with depressive symptoms among females.^41^ However, we found no significant inverse correlation between bananas and depressive symptoms in males, and a negative relationship between moderate bananas intake and depressive symptoms in females, while the OR for high bananas intake (≥118.00 g/d) was greater than 1 in females, with no statistical significance. Further studies are required to assess whether there is a difference in their relationship by gender and to evaluate the influence of different intakes.

Such divergence among the above-mentioned studies may be formed partly due to the disparity in assessment methods of vegetable and fruit intakes, the difference in estimation means of depression, classification manners of vegetable and fruit categories, covariates adjustments, and sample sizes.

The biological mechanisms for the effects of vegetable and fruit and different types of them intakes on depressive symptoms remain not completely clear, and the following are several possible mechanisms. Vegetables and fruits are rich in phytochemicals, vitamins, and minerals. The main nutrient components of different categories of them are diverse, dark-green vegetables abound with vitamin B2, magnesium, and folic acid, tomatoes are a good source of lycopene, β-carotene, and vitamin C, and berries provide abundant flavonoids.^1^ Carotenoids and vitamin C have antioxidant characteristics.^42^ Lycopene,^43^ β-carotene,^44^ flavonoids,^45^ and magnesium^46^ have anti-inflammatory effects. Vitamin B2^47^ and folic acid^47^^,^^48^ are negatively associated with the level of serum homocysteine. Vitamin C is related to the synthesis of neurotransmitters.^49^ Dietary flavonoids can inhibit apoptosis and promote neuronal survival by interacting with neuronal signaling cascades.^45^ Besides, deficiency of magnesium can lead to dysregulation of the hypothalamic-pituitary-adrenal (HPA) axis and anxiety.^50^ Depression is associated with oxidative stress,^13^ inflammation,^12^ the high level of homocysteine,^51^ scarcity of neurotransmitter serotonin^52^ or catecholamine,^53^ neurodevelopment disruptions,^54^ and abnormal HPA axis.^55^ Therefore, sufficient carotenoids,^56^ flavonoids,^25^ vitamin C,^49^ vitamin B2,^57^ folate,^58^ and magnesium^33^ may have anti-depression effects.

There are several strengths in this study. First, the current study evaluated the relationships between different categories of vegetables and fruits intakes and depressive symptoms among the general population. It may be meaningful to guide the public to choose vegetables and fruits concretely. Second, dose-response relationships of total vegetables and total fruits intakes with the risk of depressive symptoms were explored. Third, the current sample of American adults was large-sized and nationally representative, which enhanced the statistical power to obtain more precise and reliable results. Fourth, this study adjusted multiple important potential confounding factors (including demography variables, lifestyle factors, BMI, and co-morbid health conditions) that might distort the associations of vegetable and fruit intakes with depressive symptoms. Additionally, we also performed a sensitivity analysis by excluding subjects with co-morbid health conditions, and, except for bananas, other findings remained significant, suggesting the stability of these results.

However, the present study also has some limitations. First of all, the cross-sectional design was a barrier to make a causal inference. Second, the dietary information was from two nonconsecutive 24-hr dietary recalls, which might have recall bias and random variations, so it might not completely reflect the usual intake. However, validation study has revealed that 24-hr dietary recalls have significantly fewer systematic errors than food frequency questionnaires,^59^ and the most suitable evaluation method for averages and distributions of true intake in the population is repeated 24-hr dietary recalls.^60^ Third, there might be slight seasonal availability for specific types of vegetables or fruits, and certain characteristics differed between consumers and non-consumers. Although a study has shown that almost all types of vegetables and fruits were available each season in American supermarkets, and we have already adjusted these characteristics as covariates, it might be difficult to completely eliminate residual confounding effects. Besides, the assessment of outcome was not through clinical diagnosis but using the PHQ-9. Previous studies suggested that the associations of diet with depression were not completely consistent between the outcome assessed using scale and by clinical diagnosis.^61^^,^^62^ Although PHQ-9 is a useful tool in both research and clinical setting and has 88% sensitivity and specificity in the diagnosis of major depression using 10 as the cutoff value,^29^ the interpretation of the current results should still be cautious. Finally, this study could not explore dose-response relationships between different categories of vegetables and fruits intakes and depressive symptoms as a result of only a minority of participants consuming the single specific category of vegetable or fruit.

In conclusion, this cross-sectional study indicated that intakes of tomatoes and tomato mixtures, dark-green vegetables, other vegetables, berries, dried fruits, total vegetables, and total fruits were inversely related to depressive symptoms in American adults. Thus, it may be significant to advocate the consumption of vegetables and fruits, especially for the above-mentioned types, for preventing and controlling depressive symptoms. These findings need to be further ascertained via large scale prospective cohort studies.

## APPENDIX A. SUPPLEMENTARY DATA

eTable 1. Classifications of different categories of vegetables and fruits according to Food and Nutrient Databases for Dietary Studies 2007–2014

eTable 2. The classifications of covariates

eTable 3. Correlation coefficients between the first and second 24-hr dietary recalls for vegetables and fruits intakes

eTable 4. Weighted odds ratios (95% confidence intervals) for depressive symptoms across vegetables and fruits intakes stratified by gender, National Health and Nutrition Examination Survey 2007–2014

eTable 5. Weighted odds ratios (95% confidence intervals) for depressive symptoms across vegetables and fruits intakes stratified by age, National Health and Nutrition Examination Survey 2007–2014

eTable 6. Weighted odds ratios (95% confidence intervals) for depressive symptoms across vegetables and fruits intakes after removing subjects with co-morbid health conditions (diabetes/heart disease/stroke), National Health and Nutrition Examination Survey 2007–2014
