# Supplementary material for: Intakes of Specific Categories of Vegetables and Fruits Are Inversely Associated With Depressive Symptoms Among Adults
Source: J Epidemiol. 2021 Mar 5;31(3):210–9. doi: 10.2188/jea.JE20200003 (PMC7878715; doi:10.2188/jea.JE20200003)
Supplement: Supplementary file 1 [file je-31-210-s001.pdf]

eTable 1. Classifications of different categories of vegetables and fruits according to Food and Nutrient Databases for Dietary Studies 2007–2014

	**Contents included**
**Vegetables categories**	
**White potatoes and Puerto Rican starchy vegetables**	Baked, boiled, scalloped, mashed, and fried potatoes; potato chips; mixtures of potatoes as main ingredient
**Tomatoes and tomato mixtures**	Cooked and raw tomatoes; tomato juice; chili sauce, salsa, catsup, and other tomato sauces; mixtures having tomatoes as main ingredient
**Deep-yellow vegetables**	Cooked and raw deep yellow or orange vegetables for example pumpkin, winter, squash, carrots, and sweet potatoes; mixtures of deep yellow vegetables as chief ingredient
**Dark-green vegetables**	Raw and cooked romaine, broccoli, mustard and turnip greens, collards, kale, and spinach; mixtures of dark green vegetables as main component
**Other vegetables**	Raw and cooked green beans, corn, lettuce, lima beans, peas, pickles, olives, and relishes; mixtures of "other" vegetables as major ingredient
**Fruits categories**	
**Apples**	Raw and cooked apples and applesauce
**Bananas**	Raw and cooked bananas
**Berries**	Blueberries, blackberries, strawberries, raspberries, and cranberries
**Dried fruits**	Dried apricots, apples, dates, raisins, prunes, and other dried fruits
**Citrus**	Oranges and other citrus fruits
**Melons**	Honeydew melon, cantaloupe, and watermelon

eTable 2. The classifications of covariates

Covariates	Classifications
**Age, years**	20–39; 40–59; ≥60
**Gender**	Male; Female
**Race**	Mexican American; Other Hispanic; Non-Hispanic Black; Non-Hispanic White; Other race
**Marital status**	Widowed/divorced/separated/never married; Married/living with partner
**Educational level**	Above high school; High school; Below high school;
**Family income**	$20,000 and over; Under $20,000
**Body mass index (BMI)**	≥30 kg/m^2; 25 to <30 kg/m^2; <25 kg/m^2
**Work physical activity**	Vigorous; Moderate; Other
**Recreational physical activity**	Vigorous; Moderate; Other
**Smoked at least 100 cigarettes in a lifetime**	No; Yes
**Had at least 12 alcohol drinks one year**	No; Yes
**Diabetes** [a]	No; Yes
**Hypertension** [b]	No; Yes
**Heart disease** [c]	No; Yes
**Stroke** [d]	No; Yes
**Total energy intake (kcal/d)** [e]	Continuous

[a] Diabetes was identified as a level of fasting plasma (or 2-hr plasma) glucose arriving at 126 mg/dL (or 200 mg/dL) and over, level of blood glycohemoglobin (HbA1c) ≥6.5%, using anti-diabetic pills or insulin, or self-reported doctor diagnosis.

[b] Individuals whose mean diastolic (or systolic) blood pressure ≥80 mm Hg (or ≥130 mm Hg), or currently taking prescribed for high blood pressure were classified as having hypertension.

[c] Subjects who had ever been diagnosed by a doctor that they had coronary heart disease, angina pectoris, congestive heart failure or heart attack were classified as having heart disease.

[d] Participants were considered as having a stroke if they had ever been told by a doctor that they had a stroke.

[e] Total energy intake was the summation of mean daily energy intake and mean dietary energy supplement energy through two 24-hr dietary data.

eTable 3. Correlation coefficients between the first and second 24-hr dietary recalls for vegetables and fruits intakes

	**Spearman's correlation coefficient**	***P* value**
**White potatoes and Puerto Rican starchy vegetables intake (g/d)**	0.17	<0.001
**Tomatoes and tomato mixtures intake (g/d)**	0.28	<0.001
**Deep-yellow vegetables intake (g/d)**	0.43	<0.001
**Dark-green vegetables intake (g/d)**	0.29	<0.001
**Other vegetables intake (g/d)**	0.19	<0.001
**Total vegetables intake (g/d)**	0.20	<0.001
**Apples intake (g/d)**	0.39	<0.001
**Bananas intake (g/d)**	0.51	<0.001
**Berries intake (g/d)**	0.43	<0.001
**Dried fruits intake (g/d)**	0.57	<0.001
**Citrus intake (g/d)**	0.44	<0.001
**Melons intake (g/d)**	0.41	<0.001
**Total fruits intake (g/d)**	0.32	<0.001

eTable 4. Weighted odds ratios (95% confidence intervals) for depressive symptoms across vegetables and fruits intakes stratified by gender, National Health and Nutrition Examination Survey 2007–2014

	Crude	Model 1 [a]	Model 2 [b]
**Male**			
**White potatoes and Puerto Rican starchy vegetables intake (g/d) [c]**			
Group 1 (=0)	1.00 (reference)	1.00 (reference)	1.00 (reference)
Group 2 (<96.97)	0.91 (0.66–1.26)	1.07 (0.75–1.52)	1.07 (0.75–1.51)
Group 3 (≥96.97)	0.79 (0.55–1.15)	0.85 (0.58–1.27)	0.96 (0.62–1.50)
**Tomatoes and tomato mixtures intake (g/d) [c]**			
Group 1 (=0)	1.00 (reference)	1.00 (reference)	1.00 (reference)
Group 2 (<40.00)	0.61 (0.43–0.86)**	0.66 (0.46–0.95)*	0.67 (0.47–0.97)*
Group 3 (≥40.00)	0.48 (0.34–0.68)***	0.62 (0.42–0.92)*	0.67(0.44–1.01)
**Deep-yellow vegetables intake (g/d) [c]**			
Group 1 (=0)	1.00 (reference)	1.00 (reference)	1.00 (reference)
Group 2 (<51.25)	0.58 (0.23–1.50)	0.73 (0.24–2.25)	0.77 (0.25–2.35)
Group 3 (≥51.25)	0.46 (0.25–0.84)*	0.67 (0.36–1.24)	0.74 (0.40–1.38)
**Dark-green vegetables intake (g/d) [c]**			
Group 1 (=0)	1.00 (reference)	1.00 (reference)	1.00 (reference)
Group 2 (<78.00)	0.46 (0.26–0.82)*	0.66 (0.35–1.24)	0.68 (0.36–1.28)
Group 3 (≥78.00)	0.51 (0.26–1.01)	0.62 (0.31–1.26)	0.68 (0.34–1.37)
**Other vegetables intake (g/d) [c]**			
Group 1 (=0)	1.00 (reference)	1.00 (reference)	1.00 (reference)
Group 2 (<89.86)	0.74 (0.53–1.02)	1.02 (0.68–1.51)	1.05 (0.70–1.58)
Group 3 (≥89.86)	0.47 (0.34–0.65)***	0.74 (0.51–1.06)	0.85 (0.56–1.29)
**Total vegetables intake (g/d) [d]**			
Tertile 1 (<106.47)	1.00 (reference)	1.00 (reference)	
Tertile 2 (106.47 to <226.56)	0.57 (0.39–0.82)**	0.67 (0.45–1.01)	
Tertile 3 (≥226.56)	0.47 (0.34–0.65)***	0.61 (0.43–0.87)**	
**Apples intake (g/d) [c]**			
Group 1 (=0)	1.00 (reference)	1.00 (reference)	1.00 (reference)
Group 2 (<182.00)	0.71 (0.40–1.25)	0.65 (0.33–1.27)	0.69 (0.35–1.39)
Group 3 (≥182.00)	0.65 (0.37–1.12)	0.73 (0.38–1.42)	0.82 (0.43–1.59)
**Bananas intake (g/d) [c]**			
Group 1 (=0)	1.00 (reference)	1.00 (reference)	1.00 (reference)
Group 2 (<118.00)	0.54 (0.26–1.13)	0.72 (0.31–1.66)	0.76 (0.33–1.74)
Group 3 (≥118.00)	0.60 (0.39–0.93)*	0.79 (0.48–1.27)	0.85 (0.53–1.39)
**Berries intake (g/d) [c]**			
Group 1 (=0)	1.00 (reference)	1.00 (reference)	1.00 (reference)
Group 2 (<67.00)	0.39 (0.17–0.87)*	0.72 (0.31–1.67)	0.75 (0.32–1.77)
Group 3 (≥67.00)	0.33 (0.12–0.90)*	0.51 (0.18–1.51)	0.57 (0.19–1.67)
**Dried fruits intake (g/d) [c]**			
Group 1 (=0)	1.00 (reference)	1.00 (reference)	1.00 (reference)
Group 2 (<25.50)	0.32 (0.06–1.81)	0.55 (0.10–3.22)	0.56 (0.10–3.27)
Group 3 (≥25.50)	0.43 (0.21–0.89)*	0.78 (0.37–1.65)	0.83 (0.39–1.79)
**Citrus intake (g/d) [c]**			
Group 1 (=0)	1.00 (reference)	1.00 (reference)	1.00 (reference)

Group 2 (<131.00)	0.50 (0.26–0.96)*	0.60 (0.28–1.29)	0.64 (0.29–1.38)
Group 3 (≥131.00)	0.91 (0.55–1.51)	0.98 (0.57–1.71)	1.13 (0.63–2.01)
**Melons intake (g/d)** [c]			
Group 1 (=0)	1.00 (reference)	1.00 (reference)	1.00 (reference)
Group 2 (<152.00)	0.96 (0.29–3.26)	1.65 (0.43–6.31)	1.76 (0.46–6.78)
Group 3 (≥152.00)	0.64 (0.24–1.72)	0.84 (0.31–2.25)	1.03 (0.38–2.79)
**Total fruits intake (g/d)** [d]			
Tertile 1 (<78.08)	1.00 (reference)	1.00 (reference)	
Tertile 2 (78.08 to <249.00)	0.75 (0.51–1.12)	0.94 (0.61–1.46)	
Tertile 3 (≥249.00)	0.48 (0.34–0.67)***	0.64 (0.45–0.91)*	
**Female**			
**White potatoes and Puerto Rican starchy vegetables intake (g/d)** [c]			
Group 1 (=0)	1.00 (reference)	1.00 (reference)	1.00 (reference)
Group 2 (<96.97)	1.07 (0.83–1.38)	1.00 (0.78–1.29)	1.00 (0.77–1.29)
Group 3 (≥96.97)	1.25 (1.00–1.57)	1.01 (0.78–1.32)	1.07 (0.82–1.40)
**Tomatoes and tomato mixtures intake (g/d)** [c]			
Group 1 (=0)	1.00 (reference)	1.00 (reference)	1.00 (reference)
Group 2 (<40.00)	0.84 (0.68–1.03)	0.94 (0.75–1.18)	0.94 (0.75–1.18)
Group 3 (≥40.00)	0.68 (0.55–0.84)**	0.86 (0.70–1.05)	0.89 (0.72–1.11)
**Deep-yellow vegetables intake (g/d)** [c]			
Group 1 (=0)	1.00 (reference)	1.00 (reference)	1.00 (reference)
Group 2 (<51.25)	0.73 (0.50–1.06)	0.98 (0.64–1.48)	1.00 (0.65–1.52)
Group 3 (≥51.25)	0.52 (0.35–0.76)**	0.70 (0.47–1.05)	0.73 (0.49–1.09)
**Dark-green vegetables intake (g/d)** [c]			
Group 1 (=0)	1.00 (reference)	1.00 (reference)	1.00 (reference)
Group 2 (<78.00)	0.55 (0.38–0.78)**	0.67 (0.44–1.03)	0.68 (0.44–1.04)
Group 3 (≥78.00)	0.47 (0.31–0.72)**	0.61 (0.42–0.90)*	0.63 (0.44–0.91)*
**Other vegetables intake (g/d)** [c]			
Group 1 (=0)	1.00 (reference)	1.00 (reference)	1.00 (reference)
Group 2 (<89.86)	0.58 (0.46–0.74)***	0.67 (0.51–0.88)**	0.67 (0.51–0.88)**
Group 3 (≥89.86)	0.45 (0.36–0.56)***	0.58 (0.45–0.76)***	0.58 (0.44–0.76)***
**Total vegetables intake (g/d)** [d]			
Tertile 1 (<106.47)	1.00 (reference)	1.00 (reference)	
Tertile 2 (106.47 to <226.56)	0.70 (0.56–0.87)**	0.76 (0.59–0.99)*	
Tertile 3 (≥226.56)	0.57 (0.45–0.73)***	0.68 (0.51–0.90)**	
**Apples intake (g/d)** [c]			
Group 1 (=0)	1.00 (reference)	1.00 (reference)	1.00 (reference)
Group 2 (<182.00)	0.77 (0.55–1.07)	0.89 (0.61–1.29)	0.91 (0.63–1.32)
Group 3 (≥182.00)	0.62 (0.42–0.91)*	0.78 (0.48–1.27)	0.82 (0.49–1.35)
**Bananas intake (g/d)** [c]			
Group 1 (=0)	1.00 (reference)	1.00 (reference)	1.00 (reference)
Group 2 (<118.00)	0.50 (0.32–0.76)**	0.56 (0.36–0.87)*	0.58 (0.37–0.90)*
Group 3 (≥118.00)	0.94 (0.71–1.24)	1.21 (0.92–1.59)	1.27 (0.95–1.69)
**Berries intake (g/d)** [c]			
Group 1 (=0)	1.00 (reference)	1.00 (reference)	1.00 (reference)
Group 2 (<67.00)	0.38 (0.26–0.56)***	0.71 (0.48–1.06)	0.72 (0.48–1.07)
Group 3 (≥67.00)	0.29 (0.19–0.44)***	0.41 (0.25–0.68)**	0.43 (0.26–0.69)**
**Dried fruits intake (g/d)** [c]			

Group 1 (=0)	1.00 (reference)	1.00 (reference)	1.00 (reference)
Group 2 (<25.50)	0.31 (0.16–0.60)**	0.33 (0.14–0.78)*	0.34 (0.15–0.79)*
Group 3 (≥25.50)	0.59 (0.33–1.07)	0.90 (0.49–1.67)	0.92 (0.50–1.70)
**Citrus intake (g/d)** [c]			
Group 1 (=0)	1.00 (reference)	1.00 (reference)	1.00 (reference)
Group 2 (<131.00)	0.51 (0.30–0.88)*	0.67 (0.39–1.15)	0.68 (0.39–1.18)
Group 3 (≥131.00)	0.80 (0.53–1.20)	1.00 (0.60–1.67)	1.06 (0.64–1.78)
**Melons intake (g/d)** [c]			
Group 1 (=0)	1.00 (reference)	1.00 (reference)	1.00 (reference)
Group 2 (<152.00)	0.58 (0.34–1.02)	0.80 (0.43–1.50)	0.82 (0.43–1.55)
Group 3 (≥152.00)	0.64 (0.41–1.01)	0.71 (0.44–1.16)	0.78 (0.45–1.34)
**Total fruits intake (g/d)** [d]			
Tertile 1 (<78.08)	1.00 (reference)	1.00 (reference)	
Tertile 2 (78.08 to <249.00)	0.58 (0.45–0.74)***	0.71 (0.53–0.97)*	
Tertile 3 (≥249.00)	0.55 (0.44–0.69)***	0.73 (0.57–0.95)*	

[a] Model 1 adjusted for age, race, marital status, educational level, family income, body mass index, recreational physical activity, work physical activity, smoking status, alcohol consumption, hypertension, diabetes, heart disease, stroke, and total daily energy intake (continuous, kcal/d).

[b] Model 2 additionally adjusted for total vegetables intake (continuous, g/d) for subgroups of vegetables, or total fruits intake (continuous, g/d) for subgroups of fruits.

[c] Each type of vegetable or fruits intake was segmented into three categories. Participants with no consumption the specific category of vegetable were classified into group 1 (intake =0), and individuals with consumption were divided into group 2 (<median) and group 3 (≥median) based on its median intake (g/d) among the participants with consumption.

[d] Total vegetables and total fruits intake were divided into tertiles according to their distributions in the current entire study population.

* P<0.05; ** P<0.01; *** P<0.001.

eTable 5. Weighted odds ratios (95% confidence intervals) for depressive symptoms across vegetables and fruits intakes stratified by age, National Health and Nutrition Examination Survey 2007–2014

	**Crude**	**Model 1** [a]	**Model 2** [b]
**Age 20–39 years**			
**White potatoes and Puerto Rican starchy vegetables intake (g/d)** [c]			
Group 1 (=0)	1.00 (reference)	1.00 (reference)	1.00 (reference)
Group 2 (<96.97)	0.88 (0.63–1.23)	0.81 (0.58–1.14)	0.81 (0.58–1.14)
Group 3 (≥96.97)	0.98 (0.73–1.32)	0.92 (0.66–1.29)	1.06 (0.70–1.60)
**Tomatoes and tomato mixtures intake (g/d)** [c]			
Group 1 (=0)	1.00 (reference)	1.00 (reference)	1.00 (reference)
Group 2 (<40.00)	0.76 (0.54–1.06)	0.77 (0.54–1.10)	0.78 (0.55–1.12)
Group 3 (≥40.00)	0.66 (0.47–0.93)*	0.84 (0.57–1.23)	0.93 (0.63–1.36)
**Deep-yellow vegetables intake (g/d)** [c]			
Group 1 (=0)	1.00 (reference)	1.00 (reference)	1.00 (reference)
Group 2 (<51.25)	0.74 (0.43–1.26)	0.85 (0.48–1.50)	0.88 (0.49–1.59)
Group 3 (≥51.25)	0.39 (0.22–0.69)**	0.45 (0.26–0.78)**	0.49 (0.27–0.89)*
**Dark-green vegetables intake (g/d)** [c]			
Group 1 (=0)	1.00 (reference)	1.00 (reference)	1.00 (reference)
Group 2 (<78.00)	0.55 (0.31–0.96)*	0.60 (0.33–1.09)	0.61 (0.34–1.11)
Group 3 (≥78.00)	0.26 (0.12–0.56)**	0.23 (0.13–0.41)***	0.24 (0.14–0.43)***
**Other vegetables intake (g/d)** [c]			
Group 1 (=0)	1.00 (reference)	1.00 (reference)	1.00 (reference)
Group 2 (<89.86)	0.80 (0.59–1.07)	0.86 (0.60–1.23)	0.88 (0.61–1.28)
Group 3 (≥89.86)	0.55 (0.40–0.76)***	0.71 (0.48–1.04)	0.78 (0.52–1.19)
**Total vegetables intake (g/d)** [d]			
Tertile 1 (<106.47)	1.00 (reference)	1.00 (reference)	
Tertile 2 (106.47 to <226.56)	0.53 (0.37–0.76)**	0.59 (0.39–0.89)*	
Tertile 3 (≥226.56)	0.58 (0.42–0.81)**	0.72 (0.49–1.05)	
**Apples intake (g/d)** [c]			
Group 1 (=0)	1.00 (reference)	1.00 (reference)	1.00 (reference)
Group 2 (<182.00)	1.15 (0.71–1.88)	1.18 (0.62–2.26)	1.20 (0.63–2.32)
Group 3 (≥182.00)	0.80 (0.46–1.38)	1.07 (0.55–2.07)	1.09 (0.56–2.13)
**Bananas intake (g/d)** [c]			
Group 1 (=0)	1.00 (reference)	1.00 (reference)	1.00 (reference)
Group 2 (<118.00)	0.48 (0.22–1.07)	0.48 (0.20–1.16)	0.49 (0.20–1.16)
Group 3 (≥118.00)	0.73 (0.47–1.13)	1.09 (0.68–1.74)	1.11 (0.69–1.77)
**Berries intake (g/d)** [c]			
Group 1 (=0)	1.00 (reference)	1.00 (reference)	1.00 (reference)
Group 2 (<67.00)	0.32 (0.15–0.69)**	0.44 (0.20–0.95)*	0.44 (0.20–0.96)*
Group 3 (≥67.00)	0.70 (0.33–1.47)	0.94 (0.40–2.22)	0.95 (0.40–2.28)
**Dried fruits intake (g/d)** [c]			
Group 1 (=0)	1.00 (reference)	1.00 (reference)	1.00 (reference)
Group 2 (<25.50)	0.02 (0.00–0.14)***	0.02 (0.00–0.22)**	0.02 (0.00–0.22)**
Group 3 (≥25.50)	0.32 (0.14–0.74)**	0.36 (0.14–0.93)*	0.36 (0.14–0.94)*
**Citrus intake (g/d)** [c]			
Group 1 (=0)	1.00 (reference)	1.00 (reference)	1.00 (reference)
Group 2 (<131.00)	0.83 (0.34–2.03)	1.19 (0.51–2.78)	1.20 (0.51–2.81)
Group 3 (≥131.00)	0.91 (0.51–1.63)	1.06 (0.54–2.09)	1.08 (0.55–2.14)

**Melons intake (g/d) [c]**			
Group 1 (=0)	1.00 (reference)	1.00 (reference)	1.00 (reference)
Group 2 (<152.00)	0.42 (0.18–0.95)*	0.39 (0.16–0.96)*	0.39 (0.16–0.99)*
Group 3 (≥152.00)	0.80 (0.40–1.60)	0.84 (0.38–1.83)	0.86 (0.36–2.02)
**Total fruits intake (g/d) [d]**			
Tertile 1 (<78.08)	1.00 (reference)	1.00 (reference)	
Tertile 2 (78.08 to <249.00)	0.76 (0.48–1.20)	0.89 (0.53–1.51)	
Tertile 3 (≥249.00)	0.69 (0.50–0.95)*	0.89 (0.63–1.26)	
**40–59 years**			
**White potatoes and Puerto Rican starchy vegetables intake (g/d) [c]**			
Group 1 (=0)	1.00 (reference)	1.00 (reference)	1.00 (reference)
Group 2 (<96.97)	1.10 (0.81–1.49)	1.19 (0.84–1.69)	1.19 (0.84–1.69)
Group 3 (≥96.97)	0.94 (0.70–1.26)	0.99 (0.69–1.43)	1.01 (0.70–1.45)
**Tomatoes and tomato mixtures intake (g/d) [c]**			
Group 1 (=0)	1.00 (reference)	1.00 (reference)	1.00 (reference)
Group 2 (<40.00)	0.62 (0.49–0.77)***	0.73 (0.59–0.92)**	0.74 (0.59–0.92)**
Group 3 (≥40.00)	0.58 (0.45–0.75)***	0.86 (0.63–1.16)	0.87 (0.65–1.16)
**Deep-yellow vegetables intake (g/d) [c]**			
Group 1 (=0)	1.00 (reference)	1.00 (reference)	1.00 (reference)
Group 2 (<51.25)	0.70 (0.40–1.22)	0.78 (0.41–1.49)	0.79 (0.42–1.50)
Group 3 (≥51.25)	0.64 (0.42–0.98)*	0.97 (0.60–1.56)	0.98 (0.60–1.61)
**Dark-green vegetables intake (g/d) [c]**			
Group 1 (=0)	1.00 (reference)	1.00 (reference)	1.00 (reference)
Group 2 (<78.00)	0.67 (0.43–1.04)	0.85 (0.48–1.48)	0.85 (0.48–1.48)
Group 3 (≥78.00)	0.58 (0.35–0.95)*	0.72 (0.45–1.14)	0.72 (0.46–1.14)
**Other vegetables intake (g/d) [c]**			
Group 1 (=0)	1.00 (reference)	1.00 (reference)	1.00 (reference)
Group 2 (<89.86)	0.60 (0.44–0.83)**	0.72 (0.50–1.03)	0.71 (0.49–1.02)
Group 3 (≥89.86)	0.42 (0.31–0.57)***	0.60 (0.43–0.85)**	0.56 (0.39–0.81)**
**Total vegetables intake (g/d) [d]**			
Tertile 1 (<106.47)	1.00 (reference)	1.00 (reference)	
Tertile 2 (106.47 to <226.56)	0.71 (0.55–0.93)*	0.84 (0.60–1.18)	
Tertile 3 (≥226.56)	0.50 (0.38–0.66)***	0.72 (0.53–0.99)*	
**Apples intake (g/d) [c]**			
Group 1 (=0)	1.00 (reference)	1.00 (reference)	1.00 (reference)
Group 2 (<182.00)	0.84 (0.51–1.37)	0.75 (0.45–1.25)	0.79 (0.48–1.32)
Group 3 (≥182.00)	0.49 (0.32–0.76)**	0.53 (0.31–0.92)*	0.59 (0.33–1.06)
**Bananas intake (g/d) [c]**			
Group 1 (=0)	1.00 (reference)	1.00 (reference)	1.00 (reference)
Group 2 (<118.00)	0.69 (0.40–1.20)	0.69 (0.34–1.37)	0.76 (0.37–1.56)
Group 3 (≥118.00)	0.85 (0.60–1.20)	1.13 (0.74–1.73)	1.27 (0.83–1.95)
**Berries intake (g/d) [c]**			
Group 1 (=0)	1.00 (reference)	1.00 (reference)	1.00 (reference)
Group 2 (<67.00)	0.42 (0.21–0.86)*	0.82 (0.38–1.77)	0.84 (0.39–1.81)
Group 3 (≥67.00)	0.24 (0.11–0.50)***	0.38 (0.17–0.86)*	0.42 (0.19–0.93)*
**Dried fruits intake (g/d) [c]**			
Group 1 (=0)	1.00 (reference)	1.00 (reference)	1.00 (reference)
Group 2 (<25.50)	0.32 (0.12–0.86)*	0.44 (0.14–1.39)	0.45 (0.14–1.43)

Group 3 (≥25.50)	0.54 (0.26–1.12)	1.08 (0.50–2.32)	1.12 (0.51–2.45)
**Citrus intake (g/d) [c]**			
Group 1 (=0)	1.00 (reference)	1.00 (reference)	1.00 (reference)
Group 2 (<131.00)	0.40 (0.25–0.64)***	0.44 (0.25–0.78)**	0.46 (0.26–0.83)*
Group 3 (≥131.00)	0.84 (0.51–1.38)	1.04 (0.53–2.05)	1.20 (0.62–2.33)
**Melons intake (g/d) [c]**			
Group 1 (=0)	1.00 (reference)	1.00 (reference)	1.00 (reference)
Group 2 (<152.00)	0.53 (0.25–1.15)	0.87 (0.40–1.91)	0.91 (0.42–2.00)
Group 3 (≥152.00)	0.54 (0.26–1.15)	0.68 (0.31–1.51)	0.82 (0.37–1.82)
**Total fruits intake (g/d) [d]**			
Tertile 1 (<78.08)	1.00 (reference)	1.00 (reference)	
Tertile 2 (78.08 to <249.00)	0.66 (0.50–0.87)**	0.78 (0.55–1.11)	
Tertile 3 (≥249.00)	0.48 (0.36–0.66)***	0.66 (0.44–0.98)*	
**Age ≥60 years**			
**White potatoes and Puerto Rican starchy vegetables intake (g/d) [c]**			
Group 1 (=0)	1.00 (reference)	1.00 (reference)	1.00 (reference)
Group 2 (<96.97)	1.05 (0.72–1.52)	1.04 (0.68–1.60)	1.03 (0.67–1.58)
Group 3 (≥96.97)	1.05 (0.67–1.65)	0.96 (0.59–1.57)	1.10 (0.61–1.98)
**Tomatoes and tomato mixtures intake (g/d) [c]**			
Group 1 (=0)	1.00 (reference)	1.00 (reference)	1.00 (reference)
Group 2 (<40.00)	1.00 (0.66–1.53)	1.11 (0.74–1.65)	1.11 (0.75–1.65)
Group 3 (≥40.00)	0.52 (0.36–0.74)**	0.55 (0.36–0.84)**	0.59 (0.36–0.95)*
**Deep-yellow vegetables intake (g/d) [c]**			
Group 1 (=0)	1.00 (reference)	1.00 (reference)	1.00 (reference)
Group 2 (<51.25)	0.81 (0.37–1.77)	1.19 (0.50–2.82)	1.24 (0.53–2.91)
Group 3 (≥51.25)	0.43 (0.27–0.68)**	0.52 (0.31–0.86)*	0.57 (0.35–0.92)*
**Dark-green vegetables intake (g/d) [c]**			
Group 1 (=0)	1.00 (reference)	1.00 (reference)	1.00 (reference)
Group 2 (<78.00)	0.43 (0.26–0.70)**	0.48 (0.27–0.84)*	0.48 (0.27–0.85)*
Group 3 (≥78.00)	0.70 (0.41–1.20)	0.90 (0.51–1.60)	1.00 (0.58–1.71)
**Other vegetables intake (g/d) [c]**			
Group 1 (=0)	1.00 (reference)	1.00 (reference)	1.00 (reference)
Group 2 (<89.86)	0.56 (0.37–0.83)**	0.69 (0.47–0.99)*	0.70 (0.49–1.02)
Group 3 (≥89.86)	0.47 (0.31–0.71)**	0.59 (0.37–0.94)*	0.68 (0.45–1.03)
**Total vegetables intake (g/d) [d]**			
Tertile 1 (<106.47)	1.00 (reference)	1.00 (reference)	
Tertile 2 (106.47 to <226.56)	0.75 (0.52–1.10)	0.76 (0.53–1.07)	
Tertile 3 (≥226.56)	0.53 (0.35–0.78)**	0.56 (0.36–0.87)*	
**Apples intake (g/d) [c]**			
Group 1 (=0)	1.00 (reference)	1.00 (reference)	1.00 (reference)
Group 2 (<182.00)	0.60 (0.37–0.97)*	0.59 (0.36–0.98)*	0.63 (0.39–1.03)
Group 3 (≥182.00)	0.66 (0.40–1.08)	0.71 (0.39–1.29)	0.82 (0.45–1.49)
**Bananas intake (g/d) [c]**			
Group 1 (=0)	1.00 (reference)	1.00 (reference)	1.00 (reference)
Group 2 (<118.00)	0.52 (0.31–0.88)*	0.58 (0.34–0.98)*	0.63 (0.38–1.04)
Group 3 (≥118.00)	0.66 (0.45–0.97)*	0.80 (0.54–1.18)	0.89 (0.62–1.29)
**Berries intake (g/d) [c]**			
Group 1 (=0)	1.00 (reference)	1.00 (reference)	1.00 (reference)

Group 2 (<67.00)	0.51 (0.29–0.90)*	0.93 (0.50–1.72)	0.98 (0.52–1.82)
Group 3 (≥67.00)	0.19 (0.11–0.36)***	0.24 (0.12–0.48)***	0.27 (0.13–0.53)***
**Dried fruits intake (g/d)** [c]			
Group 1 (=0)	1.00 (reference)	1.00 (reference)	1.00 (reference)
Group 2 (<25.50)	0.65 (0.27–1.56)	0.62 (0.20–1.92)	0.65 (0.21–2.01)
Group 3 (≥25.50)	0.85 (0.40–1.83)	1.18 (0.53–2.65)	1.25 (0.56–2.76)
**Citrus intake (g/d)** [c]			
Group 1 (=0)	1.00 (reference)	1.00 (reference)	1.00 (reference)
Group 2 (<131.00)	0.55 (0.27–1.12)	0.55 (0.25–1.20)	0.57 (0.26–1.27)
Group 3 (≥131.00)	0.90 (0.53–1.54)	0.84 (0.46–1.50)	0.97 (0.53–1.77)
**Melons intake (g/d)** [c]			
Group 1 (=0)	1.00 (reference)	1.00 (reference)	1.00 (reference)
Group 2 (<152.00)	1.48 (0.56–3.92)	2.23 (0.69–7.26)	2.43 (0.74–7.93)
Group 3 (≥152.00)	0.83 (0.46–1.49)	0.82 (0.45–1.51)	1.09 (0.61–1.97)
**Total fruits intake (g/d)** [d]			
Tertile 1 (<78.08)	1.00 (reference)	1.00 (reference)	
Tertile 2 (78.08 to <249.00)	0.60 (0.37–0.96)*	0.71 (0.44–1.13)	
Tertile 3 (≥249.00)	0.46 (0.29-0.73)**	0.57 (0.36-0.92)*	

[a] Model 1 adjusted for gender, race, marital status, educational level, family income, body mass index, recreational physical activity, work physical activity, smoking status, alcohol consumption, hypertension, diabetes, heart disease, stroke, and total daily energy intake (continuous, kcal/d).

[b] Model 2 additionally adjusted for total vegetables intake (continuous, g/d) for subgroups of vegetables, or total fruits intake (continuous, g/d) for subgroups of fruits.

[c] Each type of vegetable or fruits intake was segmented into three categories. Participants with no consumption the specific category of vegetable were classified into group 1 (intake =0), and individuals with consumption were divided into group 2 (<median) and group 3 (≥median) based on its median intake (g/d) among the participants with consumption.

[d] Total vegetables and total fruits intake were divided into tertiles according to their distributions in the current entire study population.

* *P* <0.05; ** *P* <0.01; *** *P* <0.001.

eTable 6. Weighted odds ratios (95% confidence intervals) for depressive symptoms across vegetables and fruits intakes after removing subjects with co-morbid health conditions (diabetes/heart disease/stroke), National Health and Nutrition Examination Survey 2007–2014

	Crude	Model 1 [a]	Model 2 [b]
**White potatoes and Puerto Rican starchy vegetables intake (g/d) [c]**			
Group 1 (=0)	1.00 (reference)	1.00 (reference)	1.00 (reference)
Group 2 (<96.97)	0.95 (0.75–1.19)	0.96 (0.76–1.22)	0.96 (0.76–1.21)
Group 3 (≥96.97)	0.96 (0.75–1.22)	0.94 (0.72–1.24)	1.01 (0.76–1.34)
**Tomatoes and tomato mixtures intake (g/d) [c]**			
Group 1 (=0)	1.00 (reference)	1.00 (reference)	1.00 (reference)
Group 2 (<40.00)	0.68 (0.55–0.85)**	0.71 (0.57–0.89)**	0.72 (0.57–0.89)**
Group 3 (≥40.00)	0.61 (0.49–0.76)***	0.76 (0.60–0.98)*	0.80(0.62–1.03)
**Deep-yellow vegetables intake (g/d) [c]**			
Group 1 (=0)	1.00 (reference)	1.00 (reference)	1.00 (reference)
Group 2 (<51.25)	0.79 (0.54–1.17)	0.96 (0.62–1.50)	0.99 (0.63–1.54)
Group 3 (≥51.25)	0.62 (0.43–0.88)**	0.82 (0.58–1.16)	0.86 (0.60–1.23)
**Dark-green vegetables intake (g/d) [c]**			
Group 1 (=0)	1.00 (reference)	1.00 (reference)	1.00 (reference)
Group 2 (<78.00)	0.52 (0.37–0.71)***	0.65 (0.45–0.95)*	0.66 (0.45–0.96)*
Group 3 (≥78.00)	0.39 (0.28–0.56)***	0.47 (0.33–0.67)***	0.48 (0.34–0.69)***
**Other vegetables intake (g/d) [c]**			
Group 1 (=0)	1.00 (reference)	1.00 (reference)	1.00 (reference)
Group 2 (<89.86)	0.66 (0.53–0.82)***	0.81 (0.63–1.04)	0.81 (0.63–1.05)
Group 3 (≥89.86)	0.50 (0.40–0.63)***	0.70 (0.54–0.90)**	0.71 (0.53–0.96)*
**Total vegetables intake (g/d) [d]**			
Tertile 1 (<106.47)	1.00 (reference)	1.00 (reference)	
Tertile 2 (106.47 to <226.56)	0.63 (0.51–0.79)***	0.71 (0.56–0.90)**	
Tertile 3 (≥226.56)	0.55 (0.45–0.67)***	0.74 (0.59–0.92)**	
**Apples intake (g/d) [c]**			
Group 1 (=0)	1.00 (reference)	1.00 (reference)	1.00 (reference)
Group 2 (<182.00)	0.80 (0.54–1.17)	0.90 (0.58–1.40)	0.94 (0.60–1.45)
Group 3 (≥182.00)	0.68 (0.47–0.98)*	0.86 (0.56–1.33)	0.92 (0.60–1.41)
**Bananas intake (g/d) [c]**			
Group 1 (=0)	1.00 (reference)	1.00 (reference)	1.00 (reference)
Group 2 (<118.00)	0.49 (0.31–0.76)**	0.66 (0.40–1.10)	0.69 (0.41–1.14)
Group 3 (≥118.00)	0.81 (0.62–1.05)	1.10 (0.82–1.47)	1.16 (0.86–1.56)
**Berries intake (g/d) [c]**			
Group 1 (=0)	1.00 (reference)	1.00 (reference)	1.00 (reference)
Group 2 (<67.00)	0.46 (0.30–0.69)***	0.78 (0.51–1.20)	0.80 (0.52–1.22)
Group 3 (≥67.00)	0.36 (0.21–0.60)***	0.49 (0.28–0.86)*	0.51 (0.29–0.90)*
**Dried fruits intake (g/d) [c]**			
Group 1 (=0)	1.00 (reference)	1.00 (reference)	1.00 (reference)
Group 2 (<25.50)	0.34 (0.17–0.68)**	0.47 (0.22–0.97)*	0.48 (0.23–0.99)*
Group 3 (≥25.50)	0.50 (0.30–0.83)**	0.85 (0.50–1.45)	0.87 (0.51–1.49)
**Citrus intake (g/d) [c]**			
Group 1 (=0)	1.00 (reference)	1.00 (reference)	1.00 (reference)
Group 2 (<131.00)	0.55 (0.33–0.90)*	0.75 (0.47–1.21)	0.77 (0.48–1.24)
Group 3 (≥131.00)	0.84 (0.57–1.24)	0.99 (0.62–1.58)	1.06 (0.66–1.70)

**Melons intake (g/d)** [c]			
Group 1 (=0)	1.00 (reference)	1.00 (reference)	1.00 (reference)
Group 2 (<152.00)	0.71 (0.36–1.41)	0.95 (0.43–2.09)	0.98 (0.44–2.17)
Group 3 (≥152.00)	0.71 (0.42–1.19)	0.85 (0.50–1.46)	0.95 (0.53–1.70)
**Total fruits intake (g/d)** [d]			
Tertile 1 (<78.08)	1.00 (reference)	1.00 (reference)	
Tertile 2 (78.08 to <249.00)	0.67 (0.50–0.89)**	0.83 (0.60–1.14)	
Tertile 3 (≥249.00)	0.55 (0.45–0.68)***	0.79 (0.64–0.97)*	

[a] Model 1 adjusted for gender, age, race, marital status, educational level, family income, body mass index, recreational physical activity, work physical activity, smoking status, alcohol consumption, hypertension, and total daily energy intake (continuous, kcal/d).

[b] Model 2 additionally adjusted for total vegetables intake (continuous, g/d) for subgroups of vegetables, or total fruits intake (continuous, g/d) for subgroups of fruits.

[c] Each type of vegetable or fruits intake was segmented into three categories. Participants with no consumption the specific category of vegetable were classified into group 1 (intake =0), and individuals with consumption were divided into group 2 (<median) and group 3 (≥median) based on its median intake (g/d) among the participants with consumption.

[d] Total vegetables and total fruits intake were divided into tertiles according to their distributions in the current entire study population.

* P <0.05; ** P <0.01; *** P <0.001.
